# Generation of a Human–Mouse Chimeric Anti-Japanese Encephalitis Virus and Zika Virus Monoclonal Antibody Using CDR Grafting

**DOI:** 10.3390/microorganisms13122868

**Published:** 2025-12-17

**Authors:** Yusha Liu, Jiayi Zhang, Jiayang Zhu, Hongxia Ni, Dong Chen, Meiqing Zhang, Yuqian Fang, Cheng Ma, Shuangwei Wang, Jie Chen, Yitian Zheng, Li Chi, Lin Cai, Jinsheng Wen

**Affiliations:** 1 School of Basic Medical Sciences, Health Science Center, Ningbo University, Ningbo 315211, China; liuyusha0627@126.com (Y.L.); fhzjy1@126.com (J.Z.); 17280861660@163.com (M.Z.); 18358684390@163.com (Y.F.); 13927212172@163.com (C.M.); 13806779941@163.com (S.W.); asdfgchen@126.com (J.C.); 18968806672@163.com (Y.Z.); m15658238085@163.com (L.C.); 17322980180@163.com (L.C.); 2 Wenzhou Seventh People’s Hospital, Wenzhou 325005, China; vwfjiayizhang@163.com (J.Z.); chendong@wmu.edu.cn (D.C.); 3 Ningbo Key Laboratory of Virus Research, Ningbo Municipal Center for Disease Control and Prevention, Ningbo 315010, China; nihongxia@126.com

**Keywords:** Japanese encephalitis virus, Zika virus, monoclonal antibody, human–mouse chimeric antibody, CDR grafting

## Abstract

Japanese encephalitis (JE) caused by Japanese encephalitis virus (JEV) is a dominant arthropod-borne disease in Asian countries. However, effective antiviral treatment for JEV has not yet been established. 2H4 is a previously identified mouse monoclonal antibody (mAb) which exhibited neutralizing activity against JEV infection. Herein, we designed a novel mAb F(ab’)_2_ 2A10-2H4-CDR by transplanting the complementarity-determining regions (CDRs) of 2H4 into the corresponding regions of a murine mAb 2A10 which has high homology with human mAb. We further expressed the recombinant human–mouse chimeric mAb 2A10-2H4-CDR-hFc by linking 2A10-2H4-CDR with CH2 and CH3 domains of one human mAb. The results of indirect immunofluorescence assay and ELISA show that 2A10-2H4-CDR-hFc can recognize the E proteins of JEV and Zika virus (ZIKV), similar to its original form 2H4. Moreover, 2A10-2H4-CDR-hFc displayed neutralizing activities against JEV and ZIKV equivalent to that of 2H4 in vitro (NT_50_ value against JEV = 0.079 μg/mL versus 0.022 μg/mL, respectively; NT_50_ value against ZIKV = 1.584 μg/mL versus 0.446 μg/mL, respectively). Both 2H4 and 2A10-2H4-CDR-hFc significantly increased the survival and reduced the serum viral burden of mice challenged by JEV or ZIKV. This study successfully validates an anti-JEV and ZIKV human–mouse chimeric mAb and establishes a basis for future application of this Ab in preventing or/and treating of both JEV and ZIKV infections.

## 1. Introduction

The flavivirus genus, encompassing Japanese encephalitis virus (JEV) and Zika virus (ZIKV), poses a great global health challenges due to its neurotropic nature and potential to cause severe neurological complications [1,2]. JEV, endemic in Asia, causes viral encephalitis with a high mortality rate of about 30% [1,3]. ZIKV ever caused an outbreak in South American countries in 2015 and contributed to several million cases of ZIKV infection worldwide from 2015 to 2017 [4]. ZIKV is linked to congenital microcephaly in newborns and Guillain–Barré syndrome (GBS) in adults [5,6]. ZIKV has spread to at least 92 countries and territories worldwide and represents a significant threat to human health [2]. JEV has three approved vaccines, namely inactivated Vero cell-cultured virus-based vaccine, inactivated mouse brain-cultured virus-based vaccine, and live-attenuated JEV SA14-14-2-based vaccine [7]. However, the recent studies from our lab and another research group suggested that the antibodies (Abs) induced by JEV vaccine inoculation or viral infection may promote ZIKV infection in vitro and in vivo [8,9], implying that safe vaccines or anti-virus agents are still required to prevent JEV. In contrast, there are no approved vaccines or therapeutic medicines against ZIKV. Since JEV and ZIKV co-exist in many countries (in particular Southeast Asian countries) [10,11,12,13,14], there is an urgent need to develop universal antiviral agents against both JEV and ZIKV infections.

Despite their similar clinical manifestations, flaviviruses also share structural and antigenic similarities [15]. Their genome can be translated into three structural proteins [capsid (C), pre-membrane (prM/M), and Envelope (E) proteins] and seven non-structural proteins. The E protein is responsible for the attachment of flavivirus to target cells and is the crucial viral antigen determining the production of neutralizing Abs. The extracellular part of E protein is composed of three domains, namely domain I (DI), domain II (DII), and domain III (DIII) [16]. E protein DII (EDII) is linked to E protein DIII (EDIII) by E protein DI (EDI) and contains a fusion loop (FL) which leads to the production of flavivirus-cross-reactive Abs. EDIII has the receptor-binding domain and contributes to virus attachment. Moreover, E protein is involved in virus–endosomal membrane fusion and the release of viral nucleic acid [16,17]. Therefore, neutralizing Abs targeting E protein play an important role in preventing and treating viral infections, and are also a key indicator for evaluating the effectiveness of vaccine inoculation.

Given the strong antigen specificity and significant efficacy of monoclonal antibodies (mAbs), they have been permitted to treat different kinds of disease in the clinic. To date, over 140 mAbs approved or regulated by FDA have been widely used in the United States alone [18,19]. However, only a few mAbs were approved for the treatment of viral infectious diseases [20], although their clinical efficacy has been verified by the success of Nirsevimab and Clesrovimab in preventing respiratory syncytial virus infection [21], Ibalizumab in treating HIV infection [22], and the Tixagevimab/Cilgavimab cocktail (Evusheld) in controlling SARS-CoV-2 infection [23]. 

In the past several years, scientists identified couples of human neutralizing mAbs against ZIKV [24,25,26,27,28,29,30,31,32,33,34,35,36]. However, whether these human mAbs belong to broad-spectrum neutralizing Abs, especially their ability to neutralize JEV, has not been explored. In comparison, are only a few anti-JEV mouse or human mAb were reported [37,38,39,40]. The number of human JEV-neutralizing mAbs is low [37,41], which severely restricts the research and future application of antibody-based therapy. Our recent study identified a human anti-JEV neutralizing mAb, which exhibits cross-neutralization against ZIKV [42]. Notwithstanding these data, the Zika Expert Workgroup indicated that ZIKV vaccines and mAbs are the priority agenda for research and development [43]. In addition, further studies are needed to comprehensively profile the capacity of anti-JEV mAbs to neutralize heterologous flaviviruses like ZIKV.

CDR grafting is a technique of transplanting CDR sequences of mAbs from certain species (such as the mouse) into the corresponding regions of mAb from another species (particularly humans), resulting into an engineered mAb. Compared to de novo antibody (Ab) screening, this technique has the advantages of shorter time consumption, lower cost, and higher success rate. In the present study, in order to generate a novel mAb that is closer to human Abs, we first constructed a mouse mAb F(ab’)_2_ 2A10-2H4-CDR by grafting the CDRs of a murine mAb 2H4 into the corresponding regions of a murine mAb 2A10 which shares higher homology with human mAb than mAb 2H4. We next designed and expressed a recombinant human–mouse chimeric mAb 2A10-2H4-CDR-hFc by linking 2A10-2H4-CDR with CH2 and CH3 domains of one human mAb. The results of ELISA and indirect fluorescence assay show that both 2A10-2H4-CDR-hFc and 2H4 could recognize E proteins of JEV and ZIKV. Moreover, both 2A10-2H4-CDR-hFc and 2H4 displayed comparable neutralizing activities against JEV and ZIKV.

## 2. Materials and Methods

### 2.1. Cell Lines, Viruses, and Animals

Both Vero cells (ATCC, CCL-81) and HEK-293T cells (ATCC, CRL-11268) were purchased from American Type Culture Center (ATCC, Manassas, VA, USA) and cultured using RPMI-1640 and MEM medium with 10% Fetus bovine serum (FBS) and 1% penicillin/streptomycin, respectively. Both JEV (strain ZJ14-52) and ZIKV (strain Zhejiang04) were prepared as described in our recent publication [42] and the viral titer was presented as plaque-forming unit (PFU)/mL. 

HRP-labeled goat anti-mouse IgG H or L chain mAbs, FITC-labeled goat anti-mouse IgG H chain mAb, and FITC-labeled mouse anti-human IgG H chain mAb were purchased from Proteintech (Chicago, IL, USA). Dithiothreitol (DTT), Isopropyl-β-D-Thiogalactoside (IPTG), Methyl cellulose M450, and Polyetherimide (PEI) transfection reagent were purchased from Sigma-Aldrich (St. Louis, MI, USA), Solarbio (Beijing, China), Ourchem (Beijing, China), and GlpBio (Montclair, CA, USA), respectively. A Biotin quick labeling kit with Biotin-LC-NHS and HRP-conjugated streptavidin were purchased from Beyotime biotechnology (Shanghai, China).

Six-week-old C57BL/6 mice were obtained from the Animal Model Research Center at Nanjing University, China, and maintained in the Animal Center of Ningbo University, China. The one-day-old neonatal pups born to the females were used for experiments.

### 2.2. Expression and Purification of Viral Proteins

The extracellular parts of JEV E protein (JEV-E_406_, aa 1-406; GenBank accession no: MK558811) and ZIKV E protein (ZIKV-E_410_, aa 1-410; GenBank accession no: KX117076.1) were expressed in *E. coli* respectively, and purified as described in our recent publication [44].

### 2.3. Designment of a Human–Mouse Chimeric mAb

**Figure 1:**
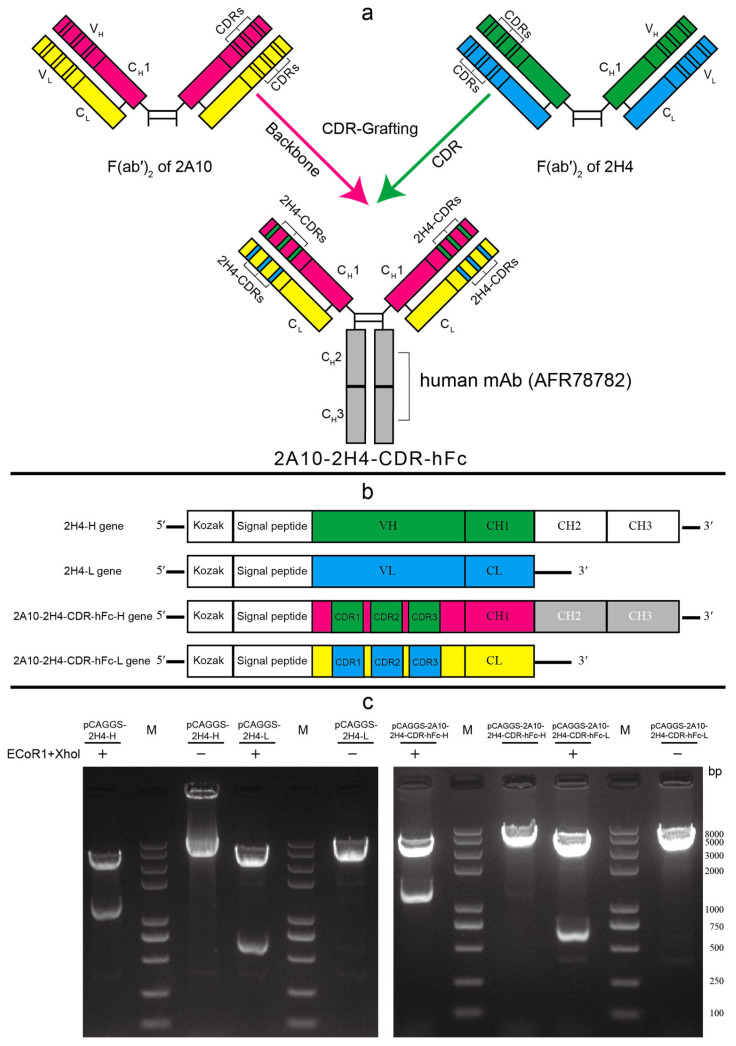
The schematic graph of constructing human–mouse chimeric monoclonal antibody. (**a**) Schematic graph of generating human–mouse chimeric mAb using CDR-grafting technique; (**b**) the schematic graph of gene sequences encoding the H or L chains of mAbs 2H4 and 2A10-2H4-CDR-hFc; (**c**) agarose gel electrophoresis of digested products of recombinant plasmids encoding L and H chains of 2H4 and 2A10-2H4-CDR-hFc.

This study attempts a new approach to partially humanize a previously reported murine anti-JEV mAb 2H4 [38]. The strategy involves first selecting a mouse mAb as backbone and then replacing the CDR sequences of this mouse mAb by the corresponding CDR sequences of 2H4, resulting in a new mouse F(ab’)_2_. Finally, this mouse F(ab’)_2_ is linked with the CH2 and CH3 domains of one human mAb to produce a human–mouse chimeric mAb. The backbone mouse mAb should match two points: (1) where it shares higher amino acid sequence homology with human mAb than mAb 2H4; (2) where the number of amino acids in each CDR region of this backbone mouse mAb is exactly identical or very close to the corresponding CDR region amino acid number of mAb 2H4. In the present study, we grafted the CDR sequences of mAb 2H4 into the corresponding sequence of a mouse mAb (2A10; Genebank accession no: 5SZF_H and 5SZF_L), which matches the above-mentioned two criteria (Table S1), and then constructed a novel mouse mAb F(ab’)_2_, which was then connected to the CH2 and CH3 sequences of one human IgG (AFR78282) to produce a human–mouse chimeric mAb 2A10-2H4-CDR-hFc (Table S2). To express this human–mouse chimeric mAb or mAb 2H4 (Table S3), we designed and synthesized two DNA sequences (Figure 1 and Table S4), which are composed of a Kozak sequence, a signal peptide gene sequence, and a light (L)/heavy (H) chain gene sequence. Homo sapiens codon-optimized DNA sequences were cloned into the eukaryotic plasmid pCAGGS to generate two recombinant plasmids: pCAGGS-2A10-2H4-CDR-hFc-H and pCAGGS-2A10-2H4-CDR-hFc-L. Similarly, the gene sequences encoding the L and H chains of murine mAb 2H4 (Table S5) were synthesized and recombinant plasmids (pCAGGS-2H4-H and pCAGGS-2H4-L) were also constructed.

### 2.4. Expression and Purification of Recombinant mAbs

The recombinant mAbs were expressed using HEK-293T cells as described in our recent publication [42]. In brief, 40 μg PEI (in 0.5 mL PBS) was added to 20 μg of recombinant plasmids (in 0.5 mL PBS) (the weight ratio of recombinant plasmid encoding H chain: recombinant plasmid encoding L chain = 1.07:0.93) and incubated for 20 min at room temperature. The mixture of plasmid and PEI was used to transfect cells for 4 h. Forty-eight hours post transfection, the cell supernatant was collected and recombinant mAbs were purified using a Pierce protein A/G agarose column (Thermo Fisher, Waltham, MA, USA). The purity of recombinant mAbs was evaluated using SDS-PAGE (in both reducing and non-reducing states) electrophoresis and Western blot (WB). The purified recombinant mAbs were used for direct ELISA, indirect immunofluorescence assay (IFA), Plaque-Reduction Neutralization Test (PRNT), and animal experiments.

### 2.5. Direct ELISA

The ability of mAbs 2H4 and 2A10-2H4-CDR-hFc to recognize JEV-E protein (JEV-E_406_) and ZIKV-E protein (ZIKV-E_410_) was evaluated by ELISA. First, both 2H4 and A10-2H4-CDR-hFc were labeled with Biotin using a Biotin quick labeling kit according to the manufacturer’s instruction. Next, ELISA plates were incubated with recombinant JEV-E_406_ or ZIKV-E_410_ proteins (2 µg/100 μL/well) at 37 °C for 1.5 h. Subsequently, the plates were incubated with 5% non-fat milk/PBS solution at 37 °C for 1.5 h. Three-fold serially diluted biotinylated 2H4 or 2A10-2H4-CDR-hFc (starting from 6 μg/mL) were added to the plates and incubated at 37 °C for 1.5 h. The plates were then incubated with HRP-conjugated streptavidin (1 µg/mL) for 1.5 h at 37 °C. Finally, the plates were incubated with freshly prepared TMB solution at room temperature for 10 min. The optical density values at 450 nm (OD_450_) were recorded using a microplate reader.

### 2.6. Immunofluorescence Assay (IFA)

Vero cells were plated on a cell crawling slice and incubated in a 37 °C/5% CO_2_ incubator for 24 h. JEV or ZIKV were used to infect Vero cells for 48 h (MOI = 1). The cells were fixed using 4% paraformaldehyde/PBS at room temperature for 1 h, and permeabilized using 0.5% Triton X-100/PBS at room temperature for 40 min. The cells were incubated with 5% bovine serum albumin (BSA)/PBS at room temperature for 1 h. The cells were incubated with mAb 2H4 or 2A10-2H4-CDR-hFc at 37 °C for 1 h. The cells were then incubated with FITC-conjugated goat anti-mouse mAb or FITC-conjugated mouse anti-human mAb at 37 °C for 1 h. The FITC-positive cells were checked using a confocal microscope.

### 2.7. Plaque-Reduction Neutralization Test (PRNT)

The ability of mAb 2H4 and 2A10-2H4-CDR-hFc to neutralize JEV or ZIKV in vitro was evaluated using a typical Vero cell-based PRNT as described in our recent publication [42]. Briefly, 3-fold serially diluted mAbs (starting from 33 μg/mL) were incubated with 50 PFU of JEV or ZIKV at 37 °C for 1 h in a 96-well cell culture plate. The mixture of mAb and virus was used to infect Vero cell in 24-well cell culture plate at 37 °C for 1 h. The cells were added with medium supplemented with 1% Methyl cellulose and maintained in the incubator for 3 or 4 days. The cells were fixed and stained with 4% paraformaldehyde/PBS and 0.5% crystal violet, respectively. The plaques were counted and the numbers were used to calculate the NT_50_ values (the minimum antibody concentration required to block half of the viral infection).

### 2.8. Mouse Experiments

The in vivo protective role of recombinant mAbs was evaluated using a 1-day-old mouse model as described in our recent publication, with little modification [42]. In brief, 10 PFU of JEV or 1000 PFU of ZIKV was injected subcutaneously (s.c.) into 1-day-old neonatal pups. After 2 h, 1 or 25 μg recombinant mAbs 2H4 or 2A10-2H4-CDR-hFc was injected s.c. into the mice. For survival study, the mouse weight and survival were recorded daily until 24 or 28 days post infection. For the tissue viral burden experiment, the mice were sacrificed 3 days post infection and the mouse sera were collected for viral load detection using PFA.

### 2.9. Viral Load Detection

The titers of each viral stock and the mouse serum viral load were detected using typical Vero cell-based PFA as described in our recent publication [42]. In brief, 3-fold serially diluted viral stock or mouse serum was used to infect cells at 37 °C for 1 h. The cells were cultured in medium supplemented with 1% Methyl cellulose for 3 or 4 days. The cells were fixed and stained with 4% paraformaldehyde/PBS and 0.5% crystal violet, respectively. The numbers of plaques in each well were counted and the viral titers in the viral stock or mouse serum were recorded as PFU/mL.

### 2.10. Statistical Analysis

The data were processed using Prism 8 software (GraphPad Software, La Jolla, CA, USA) and presented as the means ± S.E.M. Comparisons between two groups and multiple comparisons were performed by the two-tailed Mann–Whitney U test and one-way ANOVA, respectively. The survival data were processed using a log-rank test. *p* < 0.05 indicates a significant difference.

## 3. Results

### 3.1. Construction of Human–Mouse Chimeric mAb 2A10-2H4-CDR-hFc via CDR Grafting

**Figure 2:**
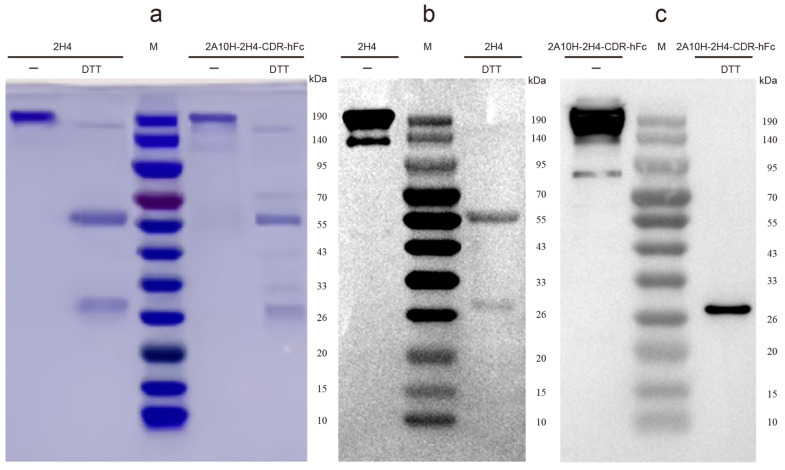
SDS-PAGE and WB results of human–mouse chimeric mAb 2A10-2H4-CDR-hFc. Both mouse mAb 2H4 and human–mouse chimeric mAb 2A10-2H4-CDR-hFc were prepared by co-transfecting the recombinant plasmids encoding the light and heavy chains into HEK293T cells. The supernatants were collected twice (3-day interval) 3 days post transfection. Recombinant mAbs were purified using Protein A/G Sepharose. The purified mAbs 2H4 and 2A10-2H4-CDR-hFc were analyzed by SDS-PAGE (**a**) and WB (**b**,**c**).

So far, cases of JEV and ZIKV have appeared in nearly a hundred countries or territories worldwide (Figure S1), so there is an impending need to identify antiviral agents or therapeutic mAbs. Previous study showed that a mouse mAb 2H4 has excellent neutralizing activity against JEV [38]. In the present study, to construct human–mouse chimeric anti-JEV mAb, we plan to search for a mouse mAb that has high amino acid sequence homology with both 2H4 and human mAb using BLAST (https://blast.ncbi.nlm.nih.gov/Blast.cgi?PROGRAM=blastp&PAGE_TYPE=BlastSearch&LINK_LOC=blasthome accessed on 12 December 2025) and design a novel mouse mAb using CDR-grafting technology. Through BLAST searching, we selected a mouse mAb 2A10 (Genebank accession no: 5SZF_H and 5SZF_L) as the backbone mAb because it has higher homology with human IgG [the highest similarity between its VH+CH1 sequence and one human IgG CHK-265 VH+CH1 (Genebank accession no: 6VYV_I) is 84.4%; the highest similarity between its L chain and one human IgG 3864-6 L chain (Genebank accession no: 8TX3_J) is 95.7%] than mouse mAb 2H4 [the highest similarity between its VH+CH1 sequence and one human IgG CHK-265 VH+CH1 sequence (Genebank accession no: 6VYV_I) is 79.8%; the highest similarity between its L chain and one human IgG FabOX108 L chain (Genebank accession no:3DGG_A) is 91.7%]. Moreover, the number of amino acid residues in the CDR regions of 2A10 H or L chains is exactly the same as or highly similar to those of 2H4 H or L chains. The comparisons of amino acid sequences between 2H4 and 2A10 are shown in Table S1. To make mAb 2H4 suitable for potential clinical application, we grafted the CDRs of 2H4 onto mAb 2A10 fragments and obtained a novel mouse mAb F(ab’)_2_ (designated as 2A10-2H4-CDR) (Figure 1). We next linked the amino acid of mAb 2A10-2H4-CDR with the CH2 and CH3 amino acid sequences of one human mAb (Genebank accession no: AFR78282) and obtained one human–mouse chimeric mAb 2A10-2H4-CDR-hFc (Figure 1, Tables S2 and S4). Subsequently, mAb 2H4 (Tables S3 and S5) and human–mouse chimeric mAb 2A10-2H4-CDR-hFc were expressed in 293T cells by co-transfection of a pair of plasmids encoding H and L chains and were purified using protein A/G agarose. Purified recombinant 2H4 and 2A10-2H4-CDR-hFc were analyzed by SDS-PAGE and WB (Figure 2). Bands of H and L chains (about 50 kDa and 25 kDa, respectively) of 2A10-2H4-CDR-hFc and 2H4 were verified, suggesting the successful production of human–mouse chimeric mAb.

### 3.2. Human–Mouse Chimeric mAb 2A10-2H4-CDR-hFc Recognizes E Proteins of JEV and ZIKV

**Figure 3:**
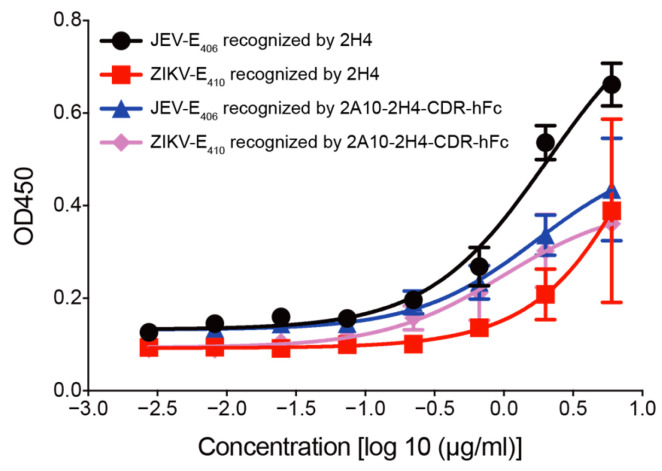
ELISA evaluation of the binding ability of recombinant mAb 2H4 and 2A10-2H4-CDR-hFc to JEV-E406 and ZIKV-E410 proteins. Recombinant JEV-E_406_ or ZIKV-E_410_ (20 μg/mL, 100 μL/well) was used to coat a high-affinity ELISA plate. Three-fold serially diluted biotinylated mAb 2H4 or 2A10-2H4-CDR-hFc (starting from 6 μg/mL) was added to ELISA plates. The ELISA plates were next incubated with HRP-conjugated streptavidin (1 µg/mL). The ELISA plates were developed with TMB and OD_450_ values of each well were measured. The experiment was conducted with three replicates and data are presented as the mean ± SEM.

**Figure 4:**
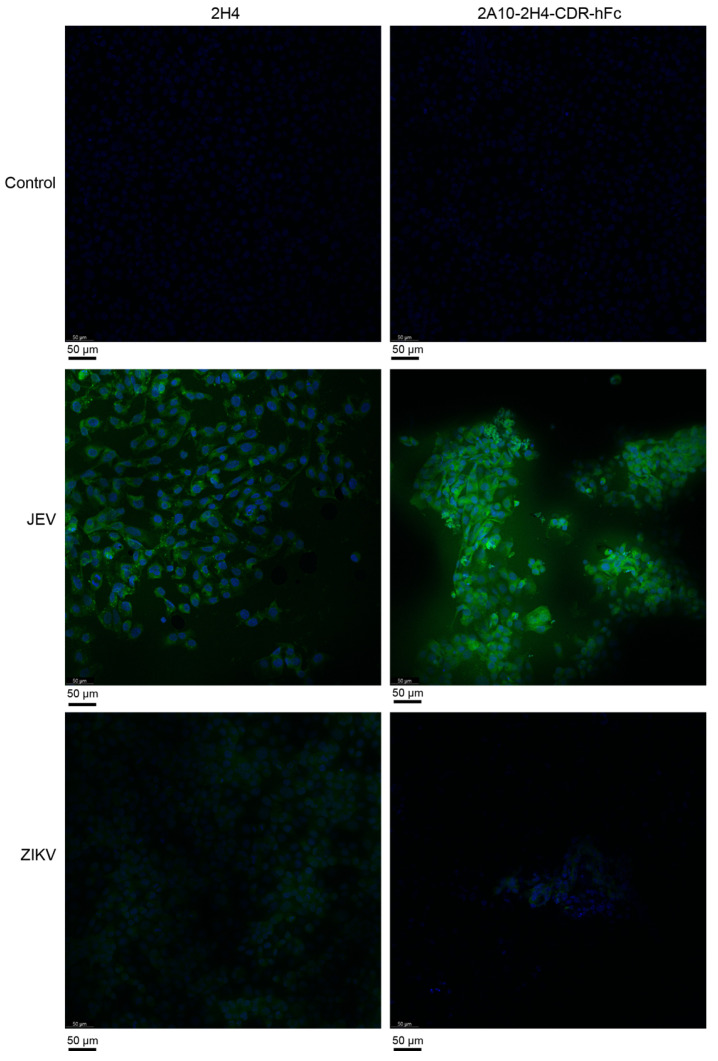
JEV and ZIKV antigens on cells recognized by human–mouse chimeric mAb 2A10-2H4-CDR-hFc. Vero cells were infected with JEV or ZIKV (MOI = 1). The cells were incubated with either mAb 2H4 or 2A10-2H4-CDR-hFc along with Hoechst 33258 (DAPI) 2 days post infection. Finally, the cells were incubated with FITC-conjugated secondary mAb. The FITC-positive cells were checked using confocal microscopy. Representative micrographs are shown. Bars, 50 μm.

Next, direct ELISA and indirect IFA were performed to explore whether human–mouse chimeric mAb 2A10-2H4-CDR could recognize JEV E protein as the original mouse mAb 2H4. The ELISA results showed that JEV-E antigen is recognized by 2H4 as well as by 2A10-2H4-CDR-hFc (Figure 3). Moreover, both 2H4 and 2A10-2H4-CDR-hFc could recognize ZIKV-E antigen. As for IFA, Vero cells were infected with JEV or ZIKV. Two days later, the expression of E proteins was detected by both 2H4 and 2A10-2H4-CDR-hFc (Figure 4). These results support the finding that human–mouse chimeric mAb 2A10-2H4-CDR-hFc retained the same antigen recognition ability as the maternal mouse mAb 2H4.

### 3.3. Human–Mouse Chimeric mAb 2A10-2H4-CDR-hFc Efficiently Neutralizes JEV and ZIKV Infections In Vitro

**Figure 5:**
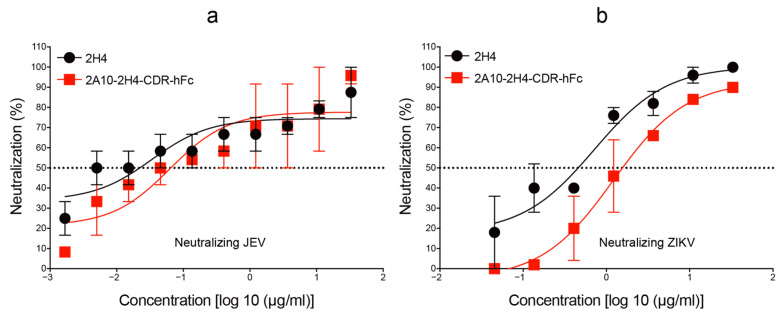
Recombinant mAb 2A10-2H4-CDR-hFc potently neutralizes JEV and ZIKV infections in vitro. The capacity of recombinant maternal mAb 2H4 and human–mouse chimeric mAb 2A10-2H4-CDR-hFc to inhibit the infection of Vero cells by JEV (**a**) or ZIKV (**b**) was tested using PRNT. Data are presented as the mean ± S.E.M.

To identify the neutralizing activities of human–mouse chimeric mAb 2A10-2H4-CDR-hFc against JEV and ZIKV, we performed typical PRNT. As shown in Figure 5, both 2A10-2H4-CDR-hFc and 2H4 inhibited JEV or ZIKV infections in a concentration-dependent manner. The NT_50_ value of 2H4 against JEV was 0.022 μg/mL in Vero cells, which is comparable to the NT_50_ of 2A10-2H4-CDR-hFc (NT_50_ = 0.079 μg/mL), indicating that the neutralizing activity of 2A10-2H4-CDR-hFc is similar to that observed for the original mouse mAb 2H4 (Figure 5). In agreement with the IFA and ELISA results, both 2H4 and 2A10-2H4-CDR-hFc could prevent ZIKV infection of Vero cells, with NT_50_ values of 0.446 μg/mL and 1.584 μg/mL, respectively. These results indicate that the human–mouse chimeric mAb 2A10-2H4-CDR-hFc retains the neutralizing ability of the maternal mAb 2H4 towards JEV and ZIKV.

### 3.4. A Single Injection of Recombinant 2A10-2H4-CDR-hFc mAb Remarkably Decreases the Mortality of Mice Challenged by JEV or ZIKV

**Figure 6:**
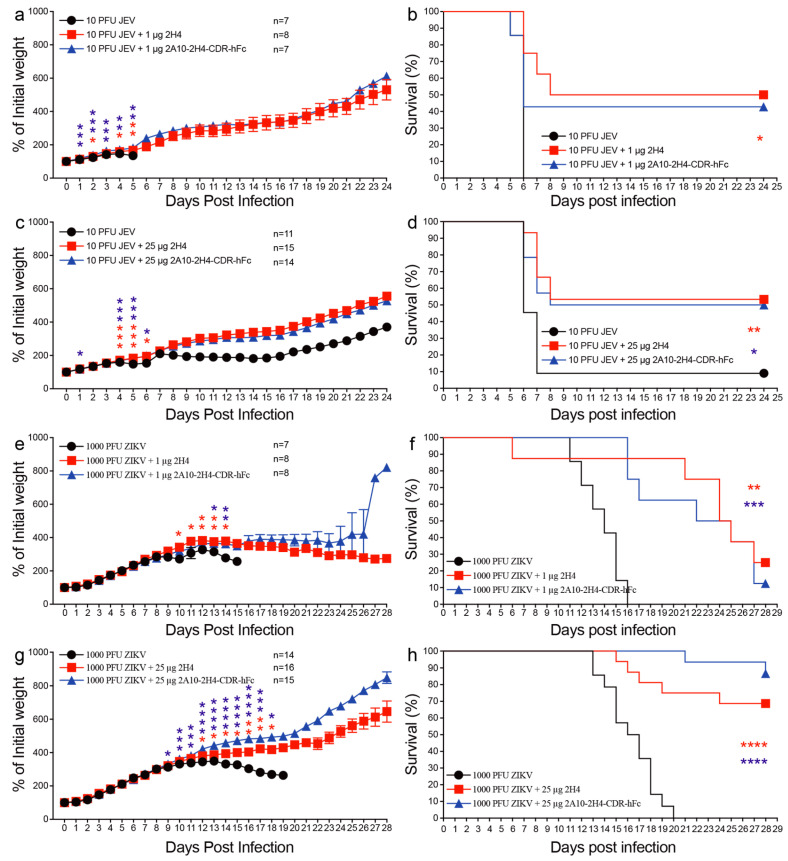
A single dose of 2A10-2H4-CDR-hFc mAb remarkably increases the percent survival of neonatal pups challenged by JEV or ZIKV. One-day-old C57BL/6 neonatal mice were injected s.c. with 10 PFU of JEV or 1000 PFU of ZIKV. After 2 h, mice were injected s.c. with 1 or 25 μg mAb (2H4 or 2A10-2H4-CDR-hFc). Mouse weight and survival were recorded daily until 24 or 28 days post viral infection. Data are expressed as the mean ± S.E.M. *, *p* < 0.05; **, *p* < 0.01; ***, *p* < 0.001; ****, *p* < 0.0001 represent significant differences, analyzed by two-tailed Mann–Whitney U test (**a**,**c**,**e**,**g**) and log-rank test (**b**,**d**,**f**,**h**), respectively. The red asterisks indicate significant differences between control group and mAb 2H4 group (1 or 25 μg); The blue asterisks indicate significant differences between control group and mAb 2A10-2H4-CDR-hFc group (1 or 25 μg).

We next explored whether this human–mouse chimeric mAb 2A10-2H4-CDR-hFc has a protective role in virus-infected mice. In this study, we chose 1-day-old neonatal mice as a mouse model to evaluate the protective effect of 2A10-2H4-CDR-hFc because neonatal mice support JEV and ZIKV infections while adult wild-type mice resist these virus infections [8]. In this study, we first injected lethal doses of JEV or ZIKV into 1-day-old neonatal pups, and then provided a single dose (1 or 25 μg) of purified 2A10-2H4-CDR-hFc or 2H4 for survival study. We selected doses of 1 or 25 μg as these correspond to 0.8 mg/kg and 20 mg/kg for 1-day-old suckling mice, which are similar to the doses used in previously published papers [42,45,46]. As shown in Figure 6a–d, >91% mice in the control groups died from JEV infection within 7 days of viral infection, but all the mice in the control groups died within 20 days of ZIKV challenge (Figure 6e–h). One μg 2H4 mAb significantly increased the percent survival of JEV-challenged mice as compared with control mice infected with JEV (50% versus 0 survival, *p* = 0.0384) (Figure 6b). The percent survival of mice administered with 25 μg 2H4 or 2A10-2H4-CDR-hFc was significantly higher than that of control mice (53.33% versus 9.09% survival, *p* = 0.0053; 50% versus 9.09% survival, *p* = 0.0202, respectively) (Figure 6d). Similarly, the percent survival of ZIKV-infected mice given 1 μg 2H4 or 2A10-2H4-CDR-hFc was also remarkably higher than that of control mice (25% versus 0 survival, *p* = 0.0018; 12.5% versus 0 survival, *p* = 0.0003, respectively) (Figure 6f). The percent survival of ZIKV-challenged mice injected with 25 μg 2H4 or 2A10-2H4-CDR-hFc was markedly higher than that of control mice infected with ZIKV too (68.75% versus 0 survival, *p* < 0.0001; 86.67% versus 0 survival, *p* < 0.0001, respectively) (Figure 6h).

In addition, application of both 2H4 and 2A10-2H4-CDR-hFc significantly attenuated the weight loss of JEV- and ZIKV-challenged mice (Figure 6a,c,e,g). These data support the finding that human–mouse chimeric mAb 2A10-2H4-CDR-hFc retains the same protection against both JEV and ZIKV infections in vivo as the maternal mAb 2H4.

### 3.5. Application of 2A10-2H4-CDR-hFc mAb Significantly Reduces the Serum Viral Load of Virus-Infected Neonatal Mice

**Figure 7:**
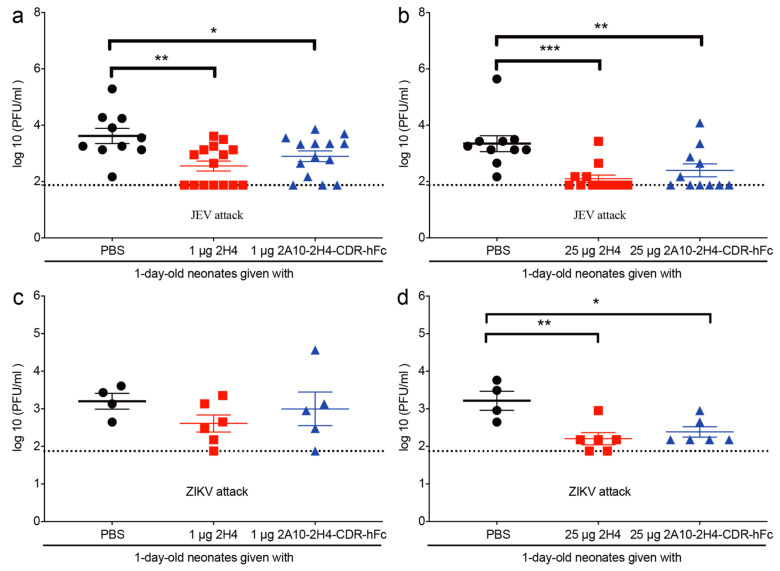
A single dose of 2A10-2H4-CDR-hFc mAb significantly reduces the tissue viral load of neonatal pups challenged by JEV or ZIKV. One-day-old C57BL/6 neonatal mice were injected s.c. with 10 PFU of JEV (**a**,**b**) or 1000 PFU of ZIKV (**c**,**d**). Mice were injected s.c. with 1 (**a**,**c**) or 25 μg (**b**,**d**) mAb (2H4 or 2A10-2H4-CDR-hFc) 2 h post infection. The mice were sacrificed 3 days post infection and the mouse sera were collected for viral load detection using PFA. The dotted lines correspond to the detection limit. Data are expressed as the mean ± S.E.M. *, *p*< 0.05; **, *p* < 0.01; ***, *p* < 0.001 by one-way ANOVA analysis.

We next studied whether 2A10-2H4-CDR-hFc could reduce mouse serum viral load. As expected, the serum viral loads of JEV-challenged mice receiving 1 μg 2H4 or 2A10-2H4-CDR-hFc were significantly lower than that of control mice (355 versus 3981 PFU/mL, *p* = 0.0022; 794 versus 3981 PFU/mL, *p* = 0.0431, respectively) (Figure 7a). Moreover, injection of 25 μg 2H4 or 2A10-2H4-CDR-hFc also significantly reduced the serum viral loads of JEV-infected mice (126 versus 2239 PFU/mL, *p* = 0.0004; 251 versus 2239 PFU/mL, *p* = 0.0085) (Figure 7b).

We next evaluated the effect of mAb 2H4 and 2A10-2H4-CDR-hFc on the serum viral burden of ZIKV-challenged mice. However, application of 1 μg 2H4 or 2A10-2H4-CDR-hFc reduced the serum viral loads of ZIKV-infected mice, but the differences are not significant (Figure 7c). As expected, 25 μg 2H4 or 2A10-2H4-CDR-hFc significantly reduced the serum viral loads of ZIKV-infected mice (158 versus 1660 PFU/mL, *p* < 0.01; 245 versus 1660 PFU/mL, *p* < 0.05) (Figure 7d).

Therefore, the above data suggest that 2A10-2H4-CDR-hFc probably protects mice against JEV and ZIKV challenge by decreasing tissue viral load.

## 4. Discussion

Due to the health risks posed by JEV and ZIKV to people in nearly 100 countries worldwide, there is an urgent need to develop more neutralizing mAbs, especially broad-spectrum mAbs with dual neutralizing effects against two viruses. Given the excellent therapeutic potential of mouse mAb 2H4 against JEV, in this study, we first adopted a CDR-grafting strategy to prepare a mouse mAb with high homology with human mAb, and then developed a human–mouse chimeric mAb. We identified that this human–mouse chimeric mAb retained the ability to neutralize JEV. We also observed that both the maternal mAb 2H4 and human–mouse chimeric mAb have neutralizing activity against ZIKV infection. These results indicate that this newly generated human–mouse chimeric mAb may have good application value in the prevention and treatment of JEV and ZIKV infections.

Among the strategies of reducing the immunogenicity of murine mAbs, CDR-grafting technology has been widely used to prepare humanized mAbs with similar functions to their maternal mAbs [47,48]. Although this technology can convert mouse mAbs into fully human mAbs (excluding CDR sequences), the antigen-binding ability of the humanized mAbs may be significantly lower than that of maternal mAb [49] because certain amino acids in the framework residues (FRs) of mAb may affect the binding affinity to antigen. Grafting the CDR sequences of maternal mouse mAbs to mouse mAbs that are highly similar to human mAbs may avoid the drawbacks of traditional CDR-grafting method, as the sequence of the recipient mouse mAb backbone region is highly similar to that of maternal mouse mAbs, which may not affect CDR exposure and antigen binding. In the present study, the mouse mAb 2A10 was chosen because it has high homology in the amino acid sequence with the human mAb and 2H4. The amino acid numbers of the six CDR sequences of 2H4 and 2A10 are completely identical or very close, which may explain why this human–mouse chimeric mAb exhibited the virus-neutralizing ability similar to that of its maternal mAb 2H4. Since the backbone mouse mAb 2A10 has higher amino acid sequence homology to human mAbs than 2H4, the engineered chimeric may induce weak immune responses when used in humans compared to the maternal mAb 2H4.

A recent study reported that 2H4 neutralizes JEV infection with an NT_50_ of 0.0012 μg/mL [38], while the present study showed that 2H4 has an NT_50_ of 0.022 μg/mL. This deviation in NT_50_ values may be caused by methodological differences: we used a 24-well culture plate for the typical PRNT in the present study, while the previous researchers performed focus-forming assay (FFA) using a 96-well culture plate [38]. Nonetheless, this study confirms that the mouse mAb 2H4 and human–mouse chimeric mAb 2A10-2H4-CDR-hFc have in vitro and in vivo neutralizing effects on both JEV and ZIKV. Their NT_50_ values (0.446 μg/mL and 1.584 μg/mL, respectively) against ZIKV are comparable to our recently identified human mAb LZY3412 and other human mAbs (Z23, ZV67, ZIKV-195, and G9E), which demonstrated NT_50_ values of 0.631 μg/mL, 0.37 μg/mL, 0.511 μg/mL, 0.6 μg/mL, and 0.963 μg/mL, respectively [24,29,34,42,45]. This provides the possibility for this human–mouse chimeric mAb to be used for the prevention and treatment of these two viruses’ infections. 

In the present study, although the human–mouse chimeric mAb prepared using CDR-grafting technique was able to neutralize JEV and ZIKV like its maternal mAb 2H4, its neutralizing activity against viruses was about 3 times lower than that of 2H4, which may affect the therapeutic efficacy of this human–mouse chimeric mAb in vivo. These data also indicate that the amino acid sequence of the backbone mAb used for making chimeric mAb may affect the exposure of the transplanted CDR sequences, thereby affecting its effectiveness. Thus, CDR grafting has inherited limitations. With the development of artificial intelligence (AI) technology, AI-assisted CDR-grafting strategies may be able to promote the display of transplanted CDR sequences and improve the function of the prepared chimeric mAbs. In addition, although we observed the protective effect of the engineered human–mouse chimeric mAb, the results should be carefully interpreted because the 1-day-old neonatal mice have an immature immune system, which may affect its susceptibility to virus infections and the therapeutic effect of mAbs. In the future, it will be necessary to use advanced animals such as adult non-human primates to evaluate the therapeutic potential of these mAbs against viral infections.

Although we did not fully humanize the mouse mAb 2H4, we believe that its ability to induce host (human) immune response after injection into the human body is weaker than that of 2H4 due to the high homology of the amino acid sequence of the human–mouse chimeric mAb 2A10-2H4-CDR-hFc with human mAbs. Moreover, this human–mouse chimeric mAb we prepared herein contains the CH2 and CH3 sequences of human IgG, retaining the functions to kill target cells by activating NK cells and the complement system. Therefore, this study is an advancement in promoting the clinical application of mouse mAb 2H4.

## 5. Conclusions

We used CDR-grafting technology to prepare a novel mouse mAb Fab carrying the 2H4 CDR sequence, and then expressed a human–mouse chimeric mAb, which maintains the ability to bind antigens and neutralize Japanese encephalitis virus. We also identified that 2H4 and human–mouse chimeric mAb 2A10-2H4-CDR-hFc have neutralizing effects on the Zika virus.

## Data Availability

The original contributions presented in this study are included in the article/Supplementary Materials. Further inquiries can be directed to the corresponding author.

## Supplementary Materials

The following supporting information can be downloaded at: https://www.mdpi.com/article/10.3390/microorganisms13122868/s1, Figure S1: The global distribution of Japanese encephalitis virus and Zika virus, Table S1: The amino acid sequences comparison of mouse mAb 2H4 and mouse mAb 2A10, Table S2: The amino acid sequences of recombinant human–mouse chimeric mAb 2A10-2H4-CDR-hFc, Table S3: The amino acid sequences of recombinant mouse mAb 2H4, Table S4: DNA sequence encoding the H or L chains of human–mouse chimeric mAb 2A10-2H4-CDR-hFc, Table S5: DNA sequence encoding the H or L chains of mouse mAb 2H4. Refs. [50,51] can be found in Supplementary Materials.
