# Supplementary material for: Generation of a Human–Mouse Chimeric Anti-Japanese Encephalitis Virus and Zika Virus Monoclonal Antibody Using CDR Grafting
Source: Microorganisms. 2025 Dec 17;13(12):2868. doi: 10.3390/microorganisms13122868 (PMC12735863; doi:10.3390/microorganisms13122868)
Supplement: Supplementary file 1 [file microorganisms-13-02868-s001.zip › microorganisms-4008644-supplementary.pdf]

Supplementary Information

Generation of a Human–Mouse Chimeric Anti-Japanese Encephalitis Virus and Zika Virus Monoclonal Antibody Using CDR Grafting

Yusha Liu [1,†], Jiayi Zhang [2,†], Jiayang Zhu [1], Hongxia Ni [3], Dong Chen [2], Meiqing Zhang [1], Yuqian Fang [1], Cheng Ma [1], Shuangwei Wang [1], Jie Chen [1], Yitian Zheng [1], Li Chi [1], Lin Cai [1] and Jinsheng Wen [1,*]

[1] School of Basic Medical Sciences, Health Science Center, Ningbo University, Ningbo 315211, China; liuyusha0627@126.com (Y.L.); fhzjy1@126.com (J.Z.); 17280861660@163.com (M.Z.); 18358684390@163.com (Y.F.); 13927212172@163.com (C.M.); 13806779941@163.com (S.W.); asdfgchen@126.com (J.C.); 18968806672@163.com (Y.Z.); m15658238085@163.com (L.C.); 17322980180@163.com (L.C.)

[2] Wenzhou Seventh People's Hospital, Wenzhou 325005, China; vwfjiayizhang@163.com (J.Z.); chendong@wmu.edu.cn (D.C.)

[3] Ningbo Key Laboratory of Virus Research, Ningbo Municipal Center for Disease Control and Prevention, Ningbo 315010, China; nihongxia@126.com

* Correspondence: wenjinsheng@nbu.edu.cn

† These authors contributed equally to this work.

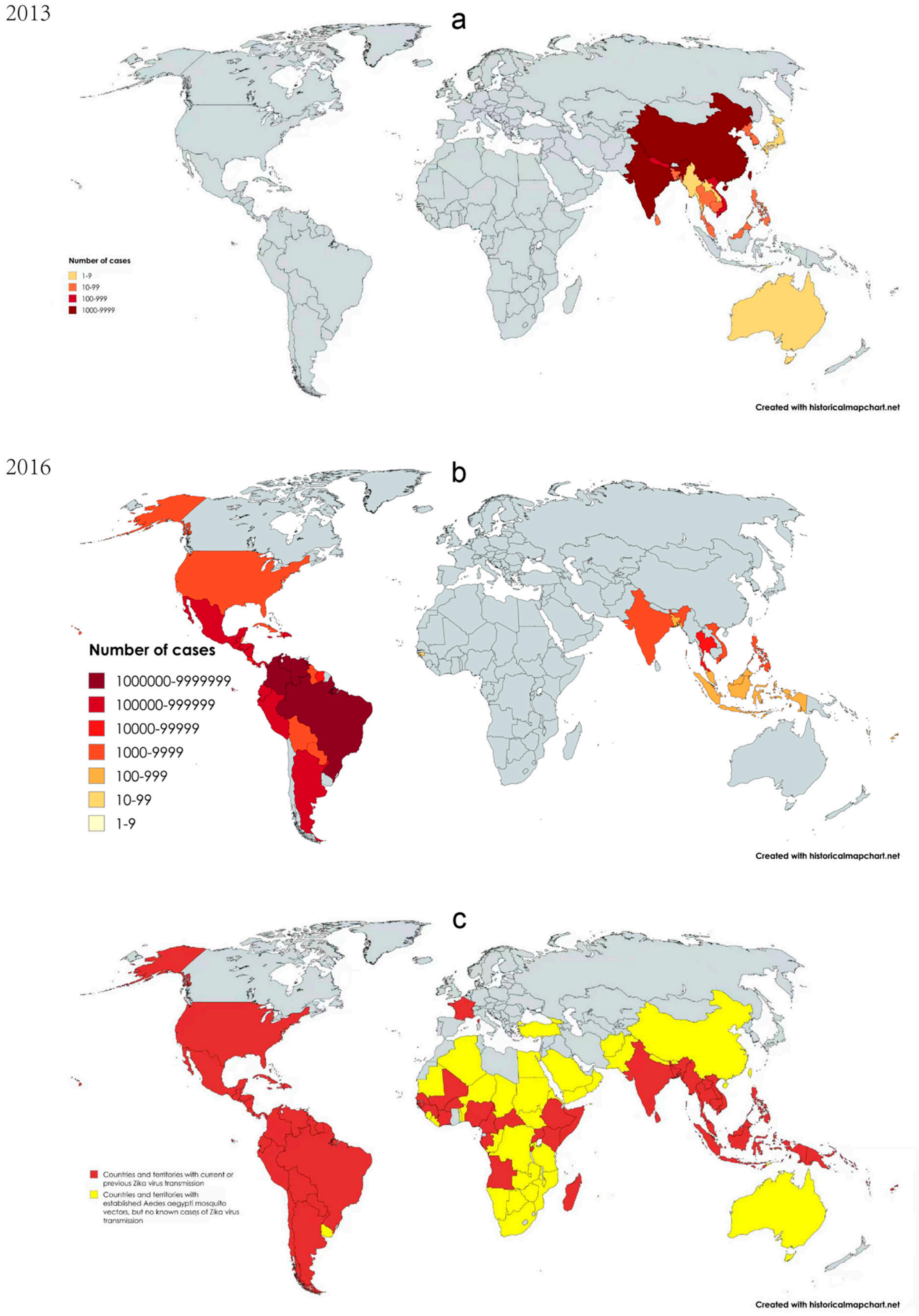

Figure S1. The global distribution of Japanese encephalitis virus and Zika virus
(a) Japanese encephalitis cases in countries and territories worldwide in 2013 (World Health Statistics, 2015, WHO)**. (b)** Zika fever cases in countries and territories worldwide in 2016. The data come from published papers[1, 2]. **(c)** Countries and territories with Zika virus transmission (Countries and territories with current or previous Zika virus transmission, WHO, data as of May 2024). The maps were created using Mapchart. World Map—Simple | Create a custom map. In: MapChart [Internet]. [cited 5 Mar 2025]. Available: https://mapchart.net/world.html

Supplementary Table S1. The amino acid sequences comparison of mouse mAbs 2H4 and 2A10

Chains	Domains	mAbs	Amino acid sequences[a]	Identity (%)	Amino acid counts
H	V	2H4	QVQLMESGPELKKPGETVKISCKASGYTFTDYSMHWVKQAPGK GLKWMGWINTGTGEPTFAADFKGRFAFSLETSASTAYLQINNLK NEDTASYFCARGVGLYGVDYWGQGTSVTVSS	82.2	118 vs 118
		2A10	QIQLVQSGPELKKPGETVKISCKASGYTFTNYGINWVKQAPGKGL KWMGWINTITEEPTFAEEFTGRFAFSLETSASTAYLQINNLKNEDT ATYFCARGSEFGRLVYWGQGASVTVSS		
	CDR1	2H4	SGYTFTDY	87.5	8 vs 8
		2A10	SGYTFTNY		
	CDR2	2H4	LKWMGWINTGTGEPTFA	88.24	17 vs 17
		2A10	LKWMGWINTITEEPTFA		
	CDR3	2H4	GVGLYGVD	12.5	8 vs 8
		2A10	GSEFGRLV		
L	V	2H4	DIVLTQSPASLAVSLGQRATISCRASQSVSTSYMHWYQQKPGQPP RLLIYLVSNLESGVPSRFSGSGSGTDFTLNIHPVEAEDEATYYCQHI RELTRSEAGPSWLEIK	56.48	108 vs 107
		2A10	DIQMTQTTSSLSASLGDRVTISCSASQGISNYLNWYQQKPDGTVK LLIFYTSTLYSGVPSRFSGSGSGTDYSLTISNLEPEDIATYYCQQYSRF PYVFGGGTKLEIK		
	CDR1	2H4	QSVSTS	33.33	6 vs 5
		2A10	QGISN		
	CDR2	2H4	PRLLIYLVSNLE	41.67	12 vs 12
		2A10	VKLLIFYTSTLY		
	CDR3	2H4	IRELTR	0	6 vs 6
		2A10	YSRFPY		

[a] Red color indicates the identical amino acid.

Supplementary Table S2. The amino acid sequences of recombinant human-mouse chimeric mAb 2A10-2H4-CDR-hFc

Chains	Domains	Amino acid sequences	Accession no
H	CDR1	SGYTFTDY	5YWF_D
	CDR2	LKWMGWINTGTGEPTFA	
	CDR3	GVGLYGVD	
L	CDR1	QSVSTS	
	CDR2	PRLLIYLVSNLE	
	CDR3	IRELTR	
H	VH	QIQLVQSGPELKKPGETVKISCKASGYTFTDYGINWVKQAPGKGLKWM GWINTGTGEPTFAEEFTGRFAFSLETSASTAYLQINNLKNEDTATYFCAR GVGLYGVDYWGQGASVTVSS	5YWF_D 5SZF_H
	CH1	AKTTAPSVYPLAPVCGDTTGSSVTLGCLVKGYFPEPVTLTWNSGSLSSGV HTFPAVLQSDLYTLSSSVTVTSSTWPSQSITCNVAHPASSTKVDKKIEPRG PT	5SZF_H
	CH2	KTHTCPPCPAPELLGGPSVFLFPPKPKDTLMISRTPEVTCVVVDVSHEDP EVKFNWYVDGVEVHNAKTKPREEQYNSTYRVVSVLTVLHQDWLNGK EYKCKVSNKALPAPIEKTISKAKGQPRE	AFR78282
	CH3	PQVYTLPPSRDELTKNQVSLTCLVKGFYPSDIAVEWESNGQPENNYKTT PPVLDSDGSFFLYSKLTVDKSRWQQGNVFSCSVMHEALHNHYTQKSLS LSPGK	
L	VL	DIQMTQTTSSLSASLGDRVTISCSASQSVSTSYLNWYQQKPDGTPRLLIYL VSNLESGVPSRFSGSGSGTDYSLTISNLEPEDIATYYCQQIRELTRVFGGGT KLEIK	5YWF_C 5SZF_L
	CL	RADAAPTVSIFPPSSEQLTSGGASVVCFLNNFYPKDINVKWKIDGSERQ NGVLNSWTDQDSKDSTYSMSSTLTLTKDEYERHNSYTCEATHKTSTSPI VKSFNRN	5SZF_L

Supplementary Table S3. The amino acid sequences of recombinant mouse mAb 2H4

Chains	Domains	Amino acid sequences	Accession no
H	CDR1	SGYTFTDY	5YWF_D
	CDR2	LKWMGWINTGTGEPTFA	
	CDR3	GVGLYGVD	
L	CDR1	QSVSTS	
	CDR2	PRLLIYLVSNLE	
	CDR3	IRELTR	
H	VH	QVQLMESGPELKKPGETVKISCKASGYTFTDYSMHWVKQAPGKGLKWMG<br>WINTGTGEPTFAADFKGRFAFSLETSASTAYLQINNLKNEDTASYFCARGVG<br>LYGVDYWGQGTSVTVSS	
	CH1	PKTTPPSVYPLAPVCGDTTGSMVTLGCLVKGYFPEPVTVTWNSGSLSSGVH<br>TFPAVLQSDLYTLSSSVTVPSSTWPSETVTCNVAHPASSTKVDKKIVPR	
	CH2	DCGCKPCICTVPEVSSVFIFPPKPKDVLTITLTPKVTCVVVDISKDDPEVQFS<br>WFVDDVEVHTAQTQPREEQFNSTFRSVSELPIMHQDWLNGKEFKCRVNSA<br>AFPAPIEKTISKTKGRPKA	QBC41012
	CH3	PQVYTIPPPKEQMAKDKVSLTCMITDFFPEDITVEWQWSGQPAENYKNTQP<br>IMDTDGSYFVYSKLNVQKSNWEAGNTFTCSVLHEGLHNHHTEKSLSHSPG<br>K	
L	VL	DIVLTQSPASLAVSLGQRATISCRASQSVSTSYMHWYQQKPGQPPRLLIYLVS<br>NLESGVPSRFSGSGSGTDFTLNIHPVEAEDEATYYCQHIRELTRSEAGPSWLE<br>IK	5YWF_C
	CL	RADAAPTVSIFPPSSEQLTSGGASVVCFLNNFYPKDINVKWKIDGSERQNG<br>VLNSWTDQDSKDSTYSMSSTLTLTKDEYERHNSYTCEATHKTSTSPIVKSFN<br>RNEC	

Supplementary Table S4. DNA sequence encoding the H or L chains of human-mouse chimeric mAb 2A10-2H4-CDR-hFc

Chains	Genes	Nucleotide sequences
H	Kozak sequence	gccacc
	Signal peptide	ATGAAGCACCTGTGGTTCTTCCTGCTCCTGGTGGCCGCCCCTAGATGGGTG CTGAGC
	VH	CAAATTCAGCTGGTGCAGAGCGGCCCCGAGCTGAAGAAGCCCGGCGAG ACCGTGAAGATCAGCTGCAAGGCTAGCGGCTACACCTTCACCGACTACG GCATCAACTGGGTGAAGCAAGCCCCCGGCAAGGGCCTGAAGTGGATGGG CTGGATCAACACCGGCACCGGCGAGCCCACCTTCGCCGAGGAGTTCACC GGCAGATTCGCCTTCAGCCTGGAGACAAGCGCTAGCACCGCCTACCTGCA GATCAACAACCTGAAGAACGAGGACACCGCCACCTACTTCTGCGCTAGA GGCGTGGGCCTGTATGGGGTGGACTACTGGGGGCAAGGCGCTAGCGTGA CCGTGAGCAGC
	CH1+CH2+CH3	GCCAAAACAACCGCCCCTAGCGTGTACCCCCTGGCCCCCGTGTGCGGCG ATACAACCGGCAGCAGCGTGACACTGGGCTGCCTCGTGAAAGGCTACTTC CCCGAGCCCGTGACCCTGACCTGGAACAGCGGCAGCCTGAGCAGCGGCG TGCACACCTTCCCCGCCGTGCTGCAGAGCGACCTGTACACCCTGAGCAGC TCCGTGACCGTGACAAGCAGCACCTGGCCTAGCCAAAGCATCACCTGCA ACGTGGCCCACCCCGCTAGCAGCACCAAGGTGGACAAGAAGATCGAGCC TAGAGGCCCCACCAAGACCCATACATGCCCTCCTTGTCCTGCTCCCGAGC TGCTCGGGGGGCCTAGCGTGTTCCTGTTCCCCCCCAAGCCCAAGGACACC CTGATGATCAGCAGAACCCCCGAGGTGACCTGCGTGGTCGTGGACGTGA GCCACGAGGACCCCGAGGTGAAGTTCAACTGGTACGTGGACGGCGTGGA GGTGCACAACGCCAAGACCAAGCCTAGAGAGGAGCAGTACAACAGCAC CTACAGAGTGGTGAGCGTGCTGACCGTGCTGCACCAAGACTGGCTGAAC GGCAAGGAGTACAAGTGCAAGGTGAGCAACAAGGCCCTGCCCGCCCCC ATCGAGAAGACCATCAGCAAGGCCAAGGGGCAGCCTAGAGAGCCCCAA GTGTACACCCTGCCCCCTAGCAGAGACGAGCTGACCAAGAACCAAGTGA GCCTGACCTGTCTGGTGAAAGGCTTCTACCCTAGCGACATCGCCGTGGAG TGGGAGAGCAACGGGCAGCCCGAGAACAACTACAAGACCACCCCCCCC GTGCTGGACAGCGACGGCAGCTTCTTCCTGTACAGCAAGCTGACCGTGGA CAAGAGCAGATGGCAGCAAGGCAACGTGTTCAGCTGCAGCGTGATGCAC GAGGCCCTGCACAACCACTACACACAGAAGAGCCTGAGCCTGAGCCCCG GCAAG
L	Kozak sequence	gccacc
	Signal peptide	ATGAAGCACCTGTGGTTCTTCCTGCTCCTGGTGGCCGCCCCTAGATGGGTG CTGAGC
	VL	GACATTCAGATGACACAGACCACAAGCAGCCTGAGCGCTAGCCTGGGCG ACAGAGTGACCATCAGCTGCAGCGCTAGCCAAAGCGTGAGCACAAGCTA CCTGAACTGGTATCAGCAGAAGCCCGACGGCACCCCTAGACTGCTGATCT ACCTGGTGTCCAATCTGGAGTCCGGCGTGCCTAGCAGATTCAGCGGCAGC GGCAGCGGCACCGACTACAGCCTGACCATCAGCAACCTGGAGCCCGAGG ACATCGCCACCTACTACTGTCAGCAGATCAGAGAGCTGACAAGAGTGTTC GGCGGGGGCACCAAGCTGGAGATCAAG
	CL	AGAGCCGACGCCGCCCCCACCGTGAGCATCTTCCCCCCTAGCAGCGAGC AGCTGACAAGCGGCGGCGCTAGCGTGGTGTGCTTCCTGAACAACTTCTAC CCCAAGGACATCAACGTGAAGTGGAAGATCGACGGCAGCGAGAGACAG AACGGCGTGCTGAACAGCTGGACCGACCAAGACAGCAAGGACAGCACC TACAGCATGAGCAGCACCCTGACCCTGACCAAGGACGAGTACGAGAGAC ACAACAGCTACACCTGCGAGGCCACCCACAAGACAAGCACAAGCCCCAT CGTGAAGAGCTTCAACAGAAAC

Supplementary Table S5. DNA sequence encoding the H or L chains of mouse mAb 2H4

Chains	Genes	Nucleotide sequences
H	Kozak sequence	gccacc
	Signal peptide	ATGAAGCACCTGTGGTTCTTCCTGCTCCTGGTGGCCGCCCCTAGATGGGTGCTGAGC
	VH	CAAGTGCAGCTGATGGAGAGCGGCCCCGAGCTGAAGAAGCCCGGCGAAACAGTG<br>AAGATCAGCTGCAAGGCTAGCGGCTACACCTTCACCGACTACAGCATGCACTGGGT<br>GAAGCAAGCCCCCGGCAAGGGCCTGAAGTGGATGGGCTGGATCAACACCGGCAC<br>CGGCGAGCCCACCTTCGCCGCCGACTTCAAGGGCAGATTCGCCTTCAGCCTGGAGA<br>CAAGCGCTAGCACCGCCTACCTGCAGATCAACAACCTGAAGAACGAGGACACCGC<br>TAGCTACTTCTGCGCTAGAGGCGTGGGCCTGTACGGCGTGGACTACTGGGGCCAAG<br>GCACCTCCGTCACAGTGAGCTCC
	CH1+CH2+CH3	CCCAAGACCACCCCTCCTAGCGTGTACCCTCTGGCCCCCGTGTGTGGCGACACCAC<br>CGGCAGCATGGTGACCCTGGGCTGCCTGGTGAAGGGCTACTTCCCCGAGCCCGTGA<br>CCGTCACATGGAATAGCGGCAGCCTGAGCAGCGGCGTGCACACCTTCCCCGCCGTG<br>CTGCAGAGCGACCTGTACACCCTGAGCAGCAGCGTGACAGTGCCTAGCAGCACCT<br>GGCCTAGCGAGACCGTGACATGCAATGTGGCCCACCCCGCTAGCAGCACCAAGGT<br>GGACAAGAAGATCGTGCCTAGAGACTGCGGCTGCAAGCCCTGCATCTGCACCGTG<br>CCCGAGGTGAGCAGCGTGTTCATCTTCCCCCCCAAGCCCAAGGACGTGCTGACCAT<br>CACCCTGACCCCCAAGGTGACCTGCGTGGTCGTGGACATCAGCAAGGACGACCCC<br>GAGGTGCAGTTCAGCTGGTTCGTGGACGACGTGGAGGTGCACACCGCTCAGACAC<br>AGCCTAGAGAGGAGCAGTTCAACAGCACCTTCAGAAGCGTGAGCGAGCTGCCCAT<br>CATGCACCAAGACTGGCTGAACGGCAAGGAGTTCAAGTGCAGAGTGAACAGCGCC<br>GCCTTCCCCGCCCCCATCGAGAAGACCATCAGCAAGACCAAGGGCAGACCCAAGG<br>CCCCCCAAGTGTACACCATCCCCCCTCCCAAGGAGCAGATGGCCAAGGACAAGGT<br>GAGCCTGACCTGCATGATCACCGACTTCTTCCCCGAGGACATCACCGTGGAGTGGC<br>AGTGGAGCGGGCAGCCCGCCGAGAACTACAAGAACACACAGCCCATCATGGACA<br>CCGACGGCAGCTACTTCGTGTACAGCAAGCTGAACGTGCAGAAGAGCAACTGGGA<br>GGCCGGCAACACCTTCACCTGCAGCGTGCTGCACGAGGGCCTGCACAACCACCAC<br>ACCGAGAAGAGCCTGAGCCACAGCCCCGGCAAG
L	Kozak sequence	gccacc
	Signal peptide	ATGAAGCACCTGTGGTTCTTCCTGCTCCTGGTGGCCGCCCCTAGATGGGTGCTGAGC
	VL	GACATCGTGCTGACACAGAGCCCCGCTAGCCTGGCCGTGAGCCTGGGGCAGAGAG<br>CCACCATCAGCTGCAGAGCTAGCCAAAGCGTGAGCACAAGCTACATGCACTGGTAT<br>CAGCAGAAGCCCGGGCAGCCCCCTAGACTGCTGATCTACCTGGTGAGCAACCTGG<br>AGAGCGGCGTGCCTAGCAGATTCAGCGGCAGCGGCAGCGGCACCGACTTCACCCT<br>GAACATCCACCCCGTGGAGGCCGAGGACGAGGCCACCTACTACTGTCAGCACATC<br>AGAGAGCTGACAAGAAGCGAGGCCGGCCCTAGCTGGCTGGAGATCAAG
	CL	AGAGCCGACGCCGCCCCCACCGTGAGCATCTTCCCCCCTAGCAGCGAGCAGCTGA<br>CAAGCGGCGGCGCTAGCGTGGTGTGCTTCCTGAACAACTTCTACCCCAAGGACATC<br>AACGTGAAGTGGAAGATCGACGGCAGCGAGAGACAGAACGGCGTGCTGAACAGC<br>TGGACCGACCAAGACAGCAAGGACAGCACCTACAGCATGAGCAGCACCCTGACC<br>CTGACCAAGGACGAGTACGAGAGACACAACAGCTACACCTGCGAGGCCACCCAC<br>AAGACAAGCACAAGCCCCATCGTGAAGAGCTTCAACAGAAACGAGTGC

References

1. Collaborators GDaI: **Global burden of 369 diseases and injuries in 204 countries and territories, 1990-2019: a systematic analysis for the Global Burden of Disease Study 2019**. *Lancet* 2020, **396**(10258):1204-1222.
2. Reasults GBoDSG: **Institute for Health Metrics and Evaluation (IHME) 2021 [cited 2021 January 12]. http://ghdx.healthdata.org/gbd-results-tool**.
